# Impaired Autoimmune T Helper 17 Cell Responses Following DNA Vaccination against Rat Experimental Autoimmune Encephalomyelitis

**DOI:** 10.1371/journal.pone.0003682

**Published:** 2008-11-10

**Authors:** Åsa Andersson, Magnus Isaksson, Judit Wefer, Anna Norling, Amilcar Flores-Morales, Fredrik Rorsman, Olle Kämpe, Robert A. Harris, Anna Lobell

**Affiliations:** 1 Department of Clinical Neuroscience, Karolinska Institute, Stockholm, Sweden; 2 Department of Medicine, University of California Los Angeles, Los Angeles, California, United States of America; 3 Department of Medical Sciences, Uppsala University, Uppsala, Sweden; 4 Department of Molecular Medicine, Karolinska Institute, Stockholm, Sweden; University Paris Sud, France

## Abstract

**Background:**

We have previously shown that vaccination with DNA encoding the encephalitogenic peptide myelin oligodendrocyte glycoprotein (MOG)_91–108_ (pMOG) suppresses MOG_91–108_-induced rat Experimental Autoimmune Encephalomyelitis (EAE), a model for human Multiple Sclerosis (MS). The suppressive effect of pMOG is dependent on inclusion of CpG DNA in the plasmid backbone and is associated with early induction of Interferon (IFN)-β.

**Principal Findings:**

In this study we examined the mechanisms underlying pMOG-induced protection. We found that in the DNA vaccinated cohort proinflammatory Interleukin (IL)-17 and IL-21 responses were dramatically reduced compared to in the control group, but that the expression of Foxp3 and Tumor Growth Factor (TGF)-β1, which are associated with regulatory T cells, was not enhanced. Moreover, genes associated with Type I IFNs were upregulated. To delineate the role of IFN-β in the protective mechanism we employed short interfering RNA (siRNA) to IFN-β in the DNA vaccine. SiRNA to IFN-β completely abrogated the protective effects of the vaccine, demonstrating that a local early elaboration of IFN-β is important for EAE protection. IL-17 responses comparable to those in control rats developed in rats injected with the IFN-β-silencing DNA vaccine.

**Conclusions:**

We herein demonstrate that DNA vaccination protects from proinflammatory Th17 cell responses during induction of EAE. The mechanism involves IFN-β as IL-17 responses are rescued by silencing of IFN-β during DNA vaccination.

## Introduction

Experimental Autoimmune Encephalomyelitis (EAE) is an animal model for the human autoimmune demyelinating disease Multiple Sclerosis (MS) [1]. Vaccination with DNA encoding myelin peptides suppresses EAE following induction with the corresponding peptide in an antigen (Ag)-specific manner [2]–[6]. Vaccination with DNA encoding myelin oligodendrocyte glycoprotein (MOG)_91–108_, pMOG, suppresses clinical signs of EAE and is associated with enhanced IFN-β expression, but MOG-specific Th1 or Th2 cell responses are not altered by pMOG vaccination [3], [7].

It has been shown that inclusion of CpG DNA within the plasmid backbone of DNA vaccines has adjuvantic properties. CpG DNAs are non-coding and unmethylated CpG motifs within the context of certain flanking bases in bacterial DNA recognized by Toll-like receptor (TLR)9 [8]. TLR9 is constitutively expressed by plasmacytoid dendritic cells (pDC) and facilitates promotion of innate immunity and type I interferon (IFN) production [8]. In pDC the adaptor molecule MyD88 binds interferon-regulatory factor-7 (IRF-7) directly, which results in high type I IFN production following TLR9 ligation, whereas ligation of TLR9 in macrophages, B cells and murine myeloid dendritic cells (mDC) leads to activation of NF-κB [9], [10]. Treatment with a DNA vaccine containing CpG DNA suppresses clinical signs of EAE in rats whereas a corresponding DNA vaccine lacking CpG DNA has no effect [3], [7], [11]. Thus the presence of CpG DNA is decisive for protective DNA vaccination against EAE.

Conversely, Ishii *et al* recently reported a TLR-independent, TANK-binding kinase-1 (TBK-1)-dependent, activation of innate and adaptive immune responses to viral proteins following DNA vaccination [12]. Plasmid DNA is a double-stranded B form of DNA which is recognized by an unknown sensor which signals via TBK-1 to induce IFN-β expression and NF-κB activation [12].

EAE was previously thought to be a purely IL-12-driven T helper (Th)1-mediated autoimmune disease [13]. However, interleukin (IL)-23 rather than IL-12 has been reported to be the critical cytokine for EAE development [14] driving encephalitogenic IL-17-producing Th cells designated Th17 [15]. Naïve CD4 T cells differentiate into Th17 cells in the presence of IL-6 and tumor growth factor (TGF)-β *in vitro* and are maintained by IL-23 [16]. It has recently been demonstrated that in fact the Th17∶Th1 ratio of infiltrating T cells determines where inflammation occurs in the CNS. T cell infiltration and inflammation in the brain only occurs when Th17 cells outnumber Th1 cells. In contrast, T cells showing a wide range of Th17∶Th1 ratios induce spinal cord parenchymal inflammation [17].

In the present study the molecular mechanisms underlying DNA vaccine-mediated protection in a rat EAE model were investigated. We demonstrate that DNA vaccination downregulates Ag-specific Th17 cell responses, and that the suppressive capability of the DNA vaccine can be abrogated by silencing IFN-β using short-interfering RNA (siRNA).

## Results

### IL-17 and IL-21 expression is abolished in central nervous system (CNS)-derived lymphocytes from DNA vaccinated rats

In our hands alterations in MOG_91–108_-specific Th1, Th2 or IL-10-producing regulatory T cell responses do not correlate with protection induced through DNA vaccination [3], [7]. However, Th17 cell responses have never been investigated in this system. We therefore examined the expression of proinflammatory cytokines which are expressed by Th17 cells following DNA vaccination. We used real-time quantitative RT-PCR (Q-PCR) to measure expression because anti-rat intracellular Abs are yet not available.

We started by examining if DNA vaccination itself induces Th17 cell responses *in vivo*. Splenocytes from DA rats treated respectively with DNA vaccines encoding MOG_91–108_, pMOG, or a control plasmid, pCI, 3 wk after DNA vaccination–but before EAE challenge - were cultured with MOG_91–108_ for 48 h to reactivate Ag-specific T cells. CD3^+^ T cells were subsequently isolated from the cultures. We failed to detect any IL-17 or IL-21 from T cells isolated from pMOG-vaccinated rats (data not included), which demonstrates that pMOG vaccination does not induce Th17 cell responses.

**Figure 1:**
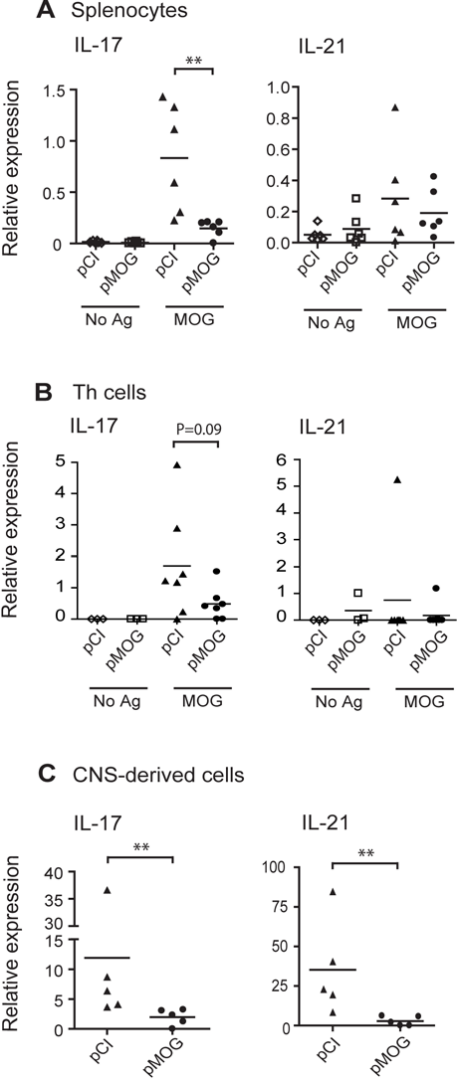
Impaired Th17 cell responses after pMOG vaccination. (A) Mean IL-17 and IL-21 mRNA expression in splenocytes after 48 h culture with medium (No Ag) or MOG_91–108_ (MOG) isolated from pMOG- or pCI-treated rats, respectively, 9 d after MOG_91–108_ immunization (*n* = 6/group). Data are representative of two separate experiments. All values are normalized to 18s rRNA. (B) Mean IL-17 and IL-21 mRNA expression in sorted CD3^+^CD4^+^ Th cells from spleen. Splenocytes were sorted after 48 h culture with medium (No Ag) or MOG_91–108_ (MOG) isolated from pMOG- or pCI-treated rats (*n* = 7/group) 11 d after MOG_91–108_ immunization. All values are normalized to GAPDH mRNA. (C) Mean IL-17 and IL-21 mRNA expression in CNS-derived lymphocytes isolated from pMOG- or pCI-treated rats (*n* = 5/group) at 11 d after MOG_91–108_ immunization. All values are normalized to GAPDH mRNA. Bars represent mean values. * *p*<0.05, ** *p*<0.01.

Thereafter, cytokine expression was assessed in splenocytes from pMOG- or pCI-treated rats during the peak of disease. Splenocytes were isolated on d 9 after MOG_91–108_ immunization and cultured for 48 h with or without MOG_91–108_. Ag-specific IL-17 mRNA expression was much lower in splenocytes from pMOG-vaccinated rats compared to in controls (*p*<0.01) (Fig. 1A). Expression of cytokines relevant for Th17 cell differentiation such as IL-21 (Fig. 1A), IL-6 and IL-1β (data not included) did not differ between the groups. In concordance with our previous findings [3], [7], [11] the expression of the Th1 cytokine IFN-γ and the anti-inflammatory cytokine IL-10 were similar in pMOG and pCI treated rats (data not included).

To confirm reduced IL-17 expression in Th17 cells, splenocytes from pMOG- or pCI-treated rats were isolated on d 11 after MOG_91–108_ immunization and cultured for 48 h with or without MOG_91–108_. CD3^+^CD4^+^ Th cells were subsequently sorted by flow cytometry. The IL-17 mRNA expression was lower in pMOG-treated rats compared to pCI-treated controls (Fig. 1B) (p = 0.09). IL-21 mRNA expression was undetectable in 6/7 Th cell samples (Fig. 1B).

Neither IFN-γ expression by CNS-derived lymphocytes nor the degree of inflammation or number of infiltrating lymphocytes within the CNS are altered by pMOG vaccination [3], but Th17 cell responses have not been investigated to date. We therefore examined Th17 cell responses in the brain and spinal cord during peak of disease by measuring IL-17 and IL-21 responses in CNS-derived lymphocytes isolated from DNA-vaccinated, pMOG-treated or pCI-treated control rats, respectively. Because infiltration of pathogenic T cells starts to occur just a few days before rats exhibit signs of disease, we isolated lymphocytes from the CNS at a timepoint when all contol rats had severe symptoms of EAE, on d 11 after immunization. Importantly, we observed abolished IL-17 (*p* = 0.008) and IL-21 (*p* = 0.008) expression in CNS-derived lymphocytes from DNA vaccinated rats compared to in controls (Fig. 1C).

We conclude that although pMOG vaccination does not affect IFN-γ production or lymphocyte infiltration into the CNS, it dramatically impairs subsequently induced MOG_91–108_-specific Th17 cell responses which correlates with protection from disease.

### Foxp3 expression is reduced in splenocytes from DNA vaccinated rats

Induced regulatory T cells (Treg) have been implicated in the protective mechanism of DNA vaccination against other organ-specific autoimmune diseases such as murine Experimental Autoimmune Uveitis [18]. Ag-specific induced TGF-β1-expressing, Foxp3^+^ Treg are primed after exposure of naïve CD4 T cells to TGF-β1 and Ag presentation in the absence of IL-6 *in vitro* [19], and induced Foxp3^+^ Treg can suppress subsequent Th17 cell responses [16], [20]. IL-10-producing Tregs are not induced by pMOG vaccination and coinjection of IL-10-coding DNA with a DNA vaccine does not increase the efficacy of the DNA vaccine [3], [7], [11] but Foxp3 expression has never been investigated in this system. We therefore examined the expression of TGF-β1 and Foxp3 which are expressed by both natural and induced Treg, following DNA vaccination.

**Figure 2:**
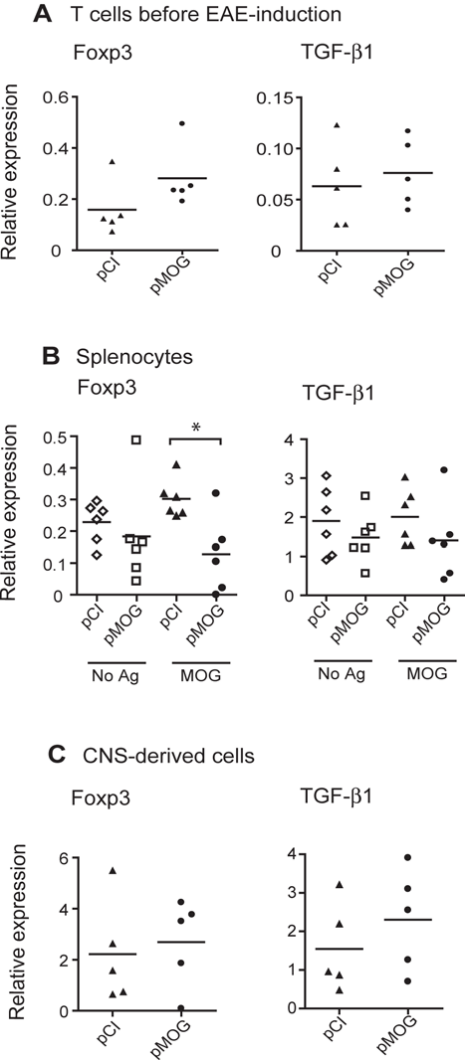
Reduced Foxp3 mRNA expression after pMOG vaccination. (A) Mean Foxp3 and TGF-β1 mRNA expression in MOG_91–108_-stimulated CD3^+^ T cells isolated from pMOG- or pCI-treated rats (*n* = 5/group) before EAE challenge. (B) Mean Foxp3 and TGF-β1 mRNA expression in splenocytes after 48 h culture with medium (No Ag) or MOG_91–108_ (MOG) isolated from pMOG- or pCI-treated rats, respectively, 9 d after MOG_91–108_ immunization (*n* = 6/group). Data are representative of two separate experiments. All values are normalized to 18s rRNA. (C) Mean Foxp3 and TGF-β1 mRNA expression in CNS-derived lymphocytes from pMOG- or pCI-treated rats (*n* = 5/group) at 11 d after MOG_91–108_ immunization. All values are normalized to GAPDH mRNA. Horisontal bars represent mean values. * *p*<0.05.

First we examined if DNA vaccination itself induced Foxp3 mRNA expression *in vivo*. Splenocytes from pMOG- or pCI-treated DA rats 3 wk after DNA vaccination – but before EAE challenge - were cultured with MOG_91–108_ for 48 h to reactivate the T cells. CD3^+^ T cells were subsequently isolated from the cultures. T cells isolated from pMOG-vaccinated rats expressed similar amounts of Foxp3 and TGF-β1 as did controls (Fig. 2A). This demonstrates that pMOG vaccination itself does not increase the Foxp3 mRNA expression in the spleen.

Splenocytes from pMOG- or pCI-treated rats during the peak of disease were then analyzed for expression of TGF-β1 and Foxp3. Splenocytes were isolated on d 9 after MOG_91–108_ immunization and cultured for 48 h with or without MOG_91–108_. Unexpectedly, Foxp3 mRNA expression was lower in MOG-stimulated splenocytes from pMOG-vaccinated rats compared to controls (*p*<0.03) (Fig. 2B). Expression of TGF-β1 (Fig. 2B) did not differ between the groups.

Finally we measured TGF-β1 and Foxp3 responses in CNS-derived lymphocytes isolated during peak of disease from DNA vaccinated, pMOG-treated or pCI-treated control rats, respectively. We failed to observe any altered expression of TGF-β1 or Foxp3 in CNS-derived lymphocytes from DNA vaccinated rats compared to controls (Fig. 2C).

Our data suggest that pMOG vaccination may reduce Foxp3 mRNA expression in the periphery.

### cDNA microarray analysis of spleens from protected DNA vaccinated rats reveals upregulation of type I IFN-associated molecules

Inclusion of CpG DNA in the plasmid backbone is required for efficient DNA vaccination against rat EAE and IFN-β expression is upregulated following DNA vaccination [3], [7], [11]. Since we failed to observe enhanced Foxp3 responses, and because IL-10 and IL-6 expression is not controlled by pMOG vaccination [3], the molecular mechanisms causing the impaired Th17 cell responses remained elusive. To identify genes that were regulated by DNA vaccination we studied the expression profiles of spleens from LEW.1AV1 rats vaccinated with pMOG or pCI during the peak of clinical EAE.

**Figure 3:**
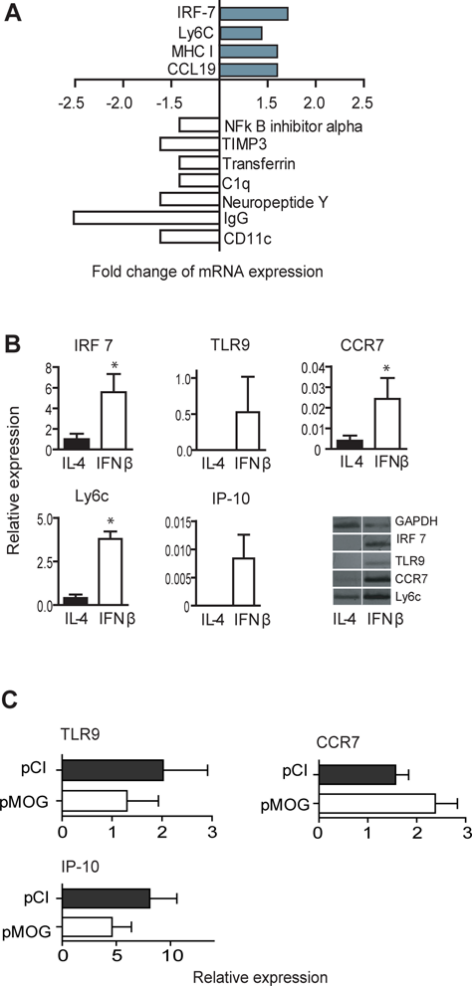
cDNA microarray analysis of DNA vaccinated rats reveals upregulated type I IFN-regulated genes. (A) Changes in gene expression of immune system-related genes in pMOG-vaccinated *vs.* pCI-vaccinated control rats at 11 d after MOG_91–108_ immunization (*n* = 6/group). Genes significantly differentially expressed as estimated using the SAM technique, which does not allow for calculations of SD or SEM. (B) Monocytes cultured with IFN-β upregulate IRF-7, CCR-7, Ly6C, TLR9 and IP-10 expression. Q-PCR analysis of the mean+/−SEM mRNA expression of relevant molecules in monocytes cultured with IL-4/GM-CSF or IFN-β/GM-CSF respectively (*n* = 4/group). (C) Mean TLR9, CCR-7 and IP-10 mRNA expression in splenocytes isolated from pMOG- or pCI-treated rats respectively 11 d after MOG_91–108_ immunization (*n* = 5/group). All values are normalized to GAPDH mRNA. Bars represent mean+/−SEM. * *p*<0.05.

Of 6240 genes printed on the array, 3390 genes were detected in the spleen; 54 transcripts were differentially expressed more than 1.4 times in DNA vaccinated rats compared to controls. Eleven genes were significantly upregulated and 43 genes were significantly downregulated following DNA vaccination (Table 1). Dataseries GSE1538 is available online at www.ncbi.nlm.nih.gov/geo. Among the upregulated genes 4/11 have reported immunological functions. Strikingly, the most upregulated genes are linked to type I IFN-induced responses and/or are pDC-associated [9], [10], [21]–[25]. A list of the differentially expressed immune system-related genes is presented in Fig. 3*A*. Differential expression of immune related genes was confirmed by Q-PCR (data not included). The differences between pMOG- and pCI-treated rats were modest and the number of differentially expressed genes was low. Most likely, the reason for this is that we compared gene expression between two MOG_91–108_ immunized groups and not between MOG_91–108_ immunized *vs* healthy rats. The cDNA microarray analysis may thus suggest a type I IFN gene signature in DNA vaccinated rats.

**Table 1:** Differentially expressed genes in DNA vaccinated rats compared to controls.

*Accession nr^a^*	*Human Locuslink ID^b^*	*Gene product*	*Microarray fold change^c^*
**Cell growth and/or maintenance^d^**			
BF281472	3925	stathmin 1/oncoprotein 18	**1.4^e^**
AW915624	6517	solute carrier family 2 (facilitated glucose transporter). member 4	**1.8**
AI070183	26471	p8 protein (candidate of metastasis 1)	−1.4
AA900048	3265	v-Ha-ras Harvey rat sarcoma viral oncogene homolog	−1.4
AA819611	3486	insulin-like growth factor binding protein 3	−1.4
AA858975	7018	Transferrine	−1.4
**Signal transduction**			
AW140799	2778	GNAS complex locus	−1.4
AW141021	4792	nuclear factor of kappa light polypeptide gene enhancer in B-cells inhibitor. alpha	−1.4
AA818383	5296	phosphoinositide-3-kinase. regulatory subunit. polypeptide 2 (p85 beta)	−1.4
AA900722	9351	solute carrier family 9. isoform 3 regulatory factor 2	−1.4
**Immune response**			
BF281806	3106	major histocompatibility complex. class I. B	**1.6**
AA965186	3665	interferon regulatory factor 7	**1.7**
AW140651	4062	lymphocyte antigen 6 complex. locus H	**1.4**
AA996885	6363	chemokine (C-C motif) ligand 19	**1.6**
AA818847	3502	immunoglobulin heavy constant gamma 3	−2.5
AW141017	3681	integrin. alpha D	−1.7
AW142249	714	complement component 1. q subcomponent. gamma polypeptide	−1.4
**Response to stress**			
AA963445	6414	selenoprotein P. plasma. 1	−1.7
**Organogenesis**			
NM012862	4256	matrix Gla protein	−2.5
AW140758	650	bone morphogenetic protein 2	−1.7
**Protein metabolism**			
AW140546	1515	cathepsin L2	−1.4
U02553	1843	dual specificity phosphatase 1	−1.4
**Neurophysiological process**			
AI045437	4852	neuropeptide Y	−1.7
**Nucleobase. nucleoside. nucleotide and nucleic acid metabolism**			
AI385189	5935	RNA binding motif protein 3	−1.7
AA819198	6943	transcription factor 21	−1.7
**Protease inhibitor activity**			
AI058471	7035	tissue factor pathway inhibitor	**1.4**
L00091	183	angiotensinogen (serine (or cysteine) proteinase inhibitor	−2
AA900912	7078	tissue inhibitor of metalloproteinase 3	−1.7
**Unassigned**			
U30789	10628	thioredoxin interacting protein	−1.9
AW144812	669	2.3-bisphosphoglycerate mutase	**1.5**
AW142974	56963	RGM domain family. member A	**1.7**
J03627	6281	S100 calcium binding protein A10	**1.4**
AW142371	1073	cofilin 2 (muscle)	−1.8
BM986386	55049	hypothetical protein FLJ20850	−1.6
AW142696	3416	insulin-degrading enzyme	−1.5
M12492	5577	protein kinase. cAMP-dependent. regulatory. type II. beta	−1.5
AW142905	115019	solute carrier family 26. member 9	−1.4

Comparison of changes in gene expression in DNA vaccinated *vs.* control rats. Genes significantly differentially expressed as estimated using the significance analysis of microarray (SAM) technique. a) Genbank accession number. b) Rat gene identities were mapped to human locuslink numbers of orthologous genes for gene categorization. c) The data are presented as ratios between the levels of test to reference cDNA that is hybridized to spotted DNA. Data represents the mean ratio of 6 arrays. d) Gene categorized using gene ontology annotation program. e) Upregulated genes are highlighted in *bold*.

In order to confirm that the genetic profile observed in the microarray analysis could be an effect of early IFN-β expression in protected rats we investigated whether the most relevant upregulated genes observed in the cDNA microarray analysis could be induced by IFN-β. We applied either IFN-β/GM-CSF or IL-4/GM-CSF *in vitro* to differentiate monocytes from LEW.1AV1 rats into pDC-like cells or mDC, respectively [26]. Monocytes cultured with IFN-β expressed significantly higher mRNA levels of IRF-7 and Ly6c, markers that were preferentially expressed in DNA vaccinated rats (Fig. 3*B*). Other markers associated with pDC, such as CC chemokine receptor (CCR)-7, TLR9 and IFN inducible protein (IP)-10, were exclusively expressed in cells cultured with IFN-β (Fig. 3*B*). Next we measured the mRNA expression of these molecules in spleens from pMOG- or pCI-treated rats during peak of disease. However, we failed to observe any upregulation of CCR7, TLR9 or IP-10 mRNA expression (Fig. 3*C*). In conclusion, DNA vacccinated rats upregulated many, but not all, of the differentially expressed genes in IFN-β-treated monocytes.

### RNA interference specific for IFN-β inhibits DNA vaccine-induced protection from EAE

**Figure 4:**
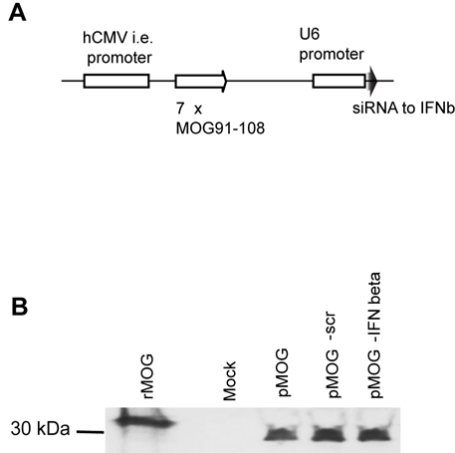
(A) Schematic portrayal of pMOG-IFNbeta contruct. A fragment containing the U6 promoter and siRNA with specificity to IFN-β was ligated downstream of the MOG_91–108_ -coding sequence of a DNA vaccine, pMOG, to form pMOG-IFNbeta. As a control for any nonspecific effect of siRNA, a non-specific siRNA sequence was ligated into pMOG to form pMOG-scr. pCI - lacking MOG_91–108_-coding DNA - was used as negative control for all DNA constructs. (B) siRNA specific for IFN-β does not alter the expression of the autoantigen MOG_91–108_ encoded by the DNA vaccine. Western blot analysis of MOG_91–108_ expression from marrow stromal cells transfected with either mock, pMOG, pMOG-scr or pMOG-IFNbeta.

The requirement of CpG DNA for the protective effect to occur [3], enhanced IFN-β mRNA expression [7] and together with the gene expression signature thus suggested that early IFN-β is involved in the protective mechanism following DNA vaccination. RNA interference was therefore employed to test *in vivo* if IFN-β is required during the initiation of the EAE-suppressive immune response following DNA vaccination. A combined DNA vaccine, pMOG-IFNbeta, was constructed that not only encoded MOG_91–108_ in tandem but also silencing siRNA specific for IFN-β (Fig. 4*A*). To exclude any effects of the siRNA *per se*, a DNA vaccine containing a scrambled, non-specific siRNA (pMOG-scr), was constructed. The scrambled siRNA was identical to the specific siRNA in bp content. We swapped the position of two bp in three locations to generate the scrambled siRNA. We first tested if expression of the encephalitogenic peptide MOG_91–108_ by the DNA vaccine was affected by siRNA by assessing MOG_91–108_ protein levels in the supernatants from DNA vaccine-transfected rat marrow stromal cells [27]. Western blot analyses revealed that MOG_91–108_ production was not affected by the siRNA (Fig. 4*B*).

**Figure 5:**
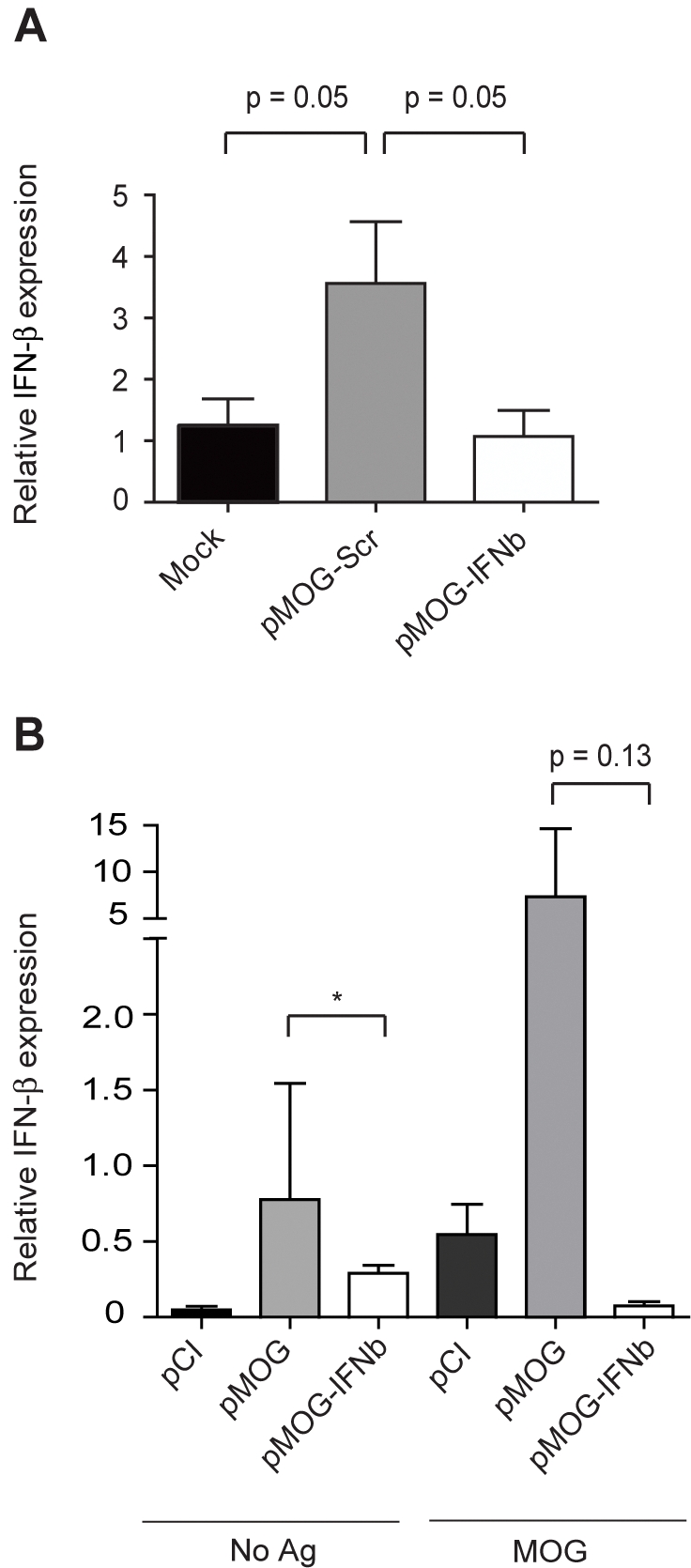
siRNA specific for IFN-β silence the mRNA expression of IFN-β. (A) Mean IFN-β mRNA expression in rat splenocytes 24 h after transfection with either mock, pMOG-scr or pMOG-IFNbeta (*n* = 6/group). All values are normalized to 18S rRNA. (*p* = 0.05). (B) siRNA specific for IFN-β dampens the mRNA expression of IFN-β after EAE challenge. Mean IFN-β mRNA expression in splenocytes after 48 h culture with medium (No Ag) or MOG_91–108_ (MOG) isolated from pMOG-, pMOG-IFNbeta- or pCI-treated rats, respectively, 11 d after MOG_91–108_ immunization (*n* = 6/group). All values are normalized to GAPDH mRNA. Bars represent mean+/−SEM . * *p*<0.05.

Next we assessed the silencing capability of the siRNA construct in pMOG-IFN-beta-, pMOG-Scr- or Mock-transfected splenocytes from untreated DA rats. IFN-β mRNA expression was induced in cells transfected with pMOG-Scr compared to Mock-transfected cells (p = 0.05) (Fig. 5A). Importantly, IFN-β mRNA expression was much lower in cells transfected with pMOG-IFNbeta compared to cells transfected with pMOG-Scr (p = 0.05) (Fig. 5A). We then analysed the IFN-β mRNA expression in splenocytes after 48 h culture with medium or MOG_91–108_ isolated from pMOG-, pMOG-IFNbeta- or pCI-treated rats, respectively, 11 d after MOG_91–108_ immunization. In contrast to pMOG, pMOG-IFNbeta failed to induce IFN-β mRNA expression in splenocytes (Fig. 5B). These data suggest that pMOG-IFNbeta silences IFN-β expression *in vitro* and *in vivo*.

**Figure 6:**
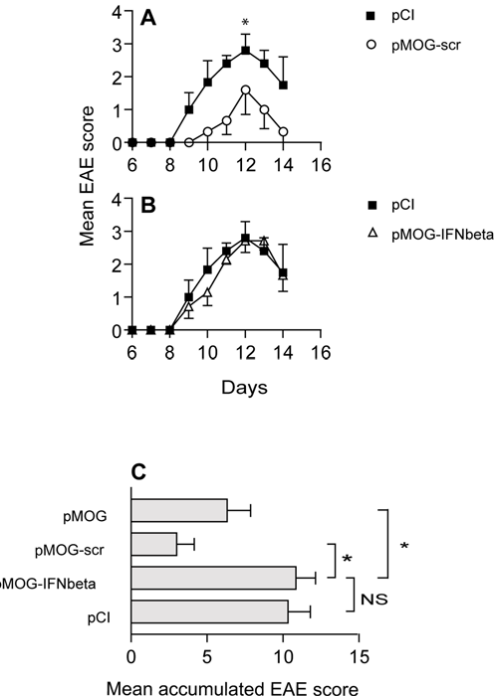
IFN-β is required for the protective effect to occur after DNA vaccination against EAE. (A) Mean daily EAE score+/−SEM (*n* = 5–8) for pMOG-scr (circles) or pCI (squares), and (B) pMOG-IFNbeta (triangles) or pCI (squares). (C) Nonspecific siRNA does not affect the suppressive effect of the DNA vaccine. The mean accumulated EAE score in pMOG-, pMOG-scr-, pCI- or pMOG-IFNbeta-treated LEW.1AV1 rats. The mean accumulated EAE score+/−SEM (*n* = 5–8/group). Data are representative of four separate experiments with the same results. A and B depicts the EAE score for rats from the same experiment. * *p*<0.05.

pMOG-IFNbeta was then tested *in vivo* for its ability to suppress EAE induced with MOG_91–108_ relative to (a) a DNA vaccine containing a non-specific siRNA (pMOG-scr), (b) a suppressive DNA vaccine (pMOG) [3] and (c) a control DNA (pCI). DNA vaccines and control DNA were injected into LEW.1AV1 rats 3-to-5 wks before EAE induction with MOG_91–108_ in CFA. Treatment with either pMOG-scr or pMOG protected the rats from EAE compared to pCI-treated controls (Fig. 6, A and C). In contrast, the DNA vaccine containing siRNA specific for IFN-β (pMOG-IFNbeta) failed to suppress clinical symptoms of EAE compared to pCI-treated controls (Fig. 6, B and C). In 4/4 experiments addition of IFN-β-specific siRNA to the DNA vaccine completely inhibited its disease-suppressive capability. The results were similar in DA rats (data not included). Thus DNA vaccine-induced IFN-β is essential for the protective effect to occur.

As the addition of siRNA itself may alter the efficacy of the pMOG construct we wanted to control for the effect of the non-specific siRNA encoded by pMOG-scr on the suppressive capability of the DNA vaccine. EAE progression and severity was compared between rats that had received the protective DNA vaccine pMOG with rats that had received pMOG-scr. No alterations in the mean daily EAE score or the mean accumulated EAE score could be determined between pMOG and pMOG-scr-treated rats (Fig. 6C), and this demonstrates that loss of protection in pMOG-IFNbeta-treated rats is specifically due to silencing of IFN-β and not due to the construct design.

### Impaired IL-17 responses following DNA vaccination are mediated by IFN-β

**Figure 7:**
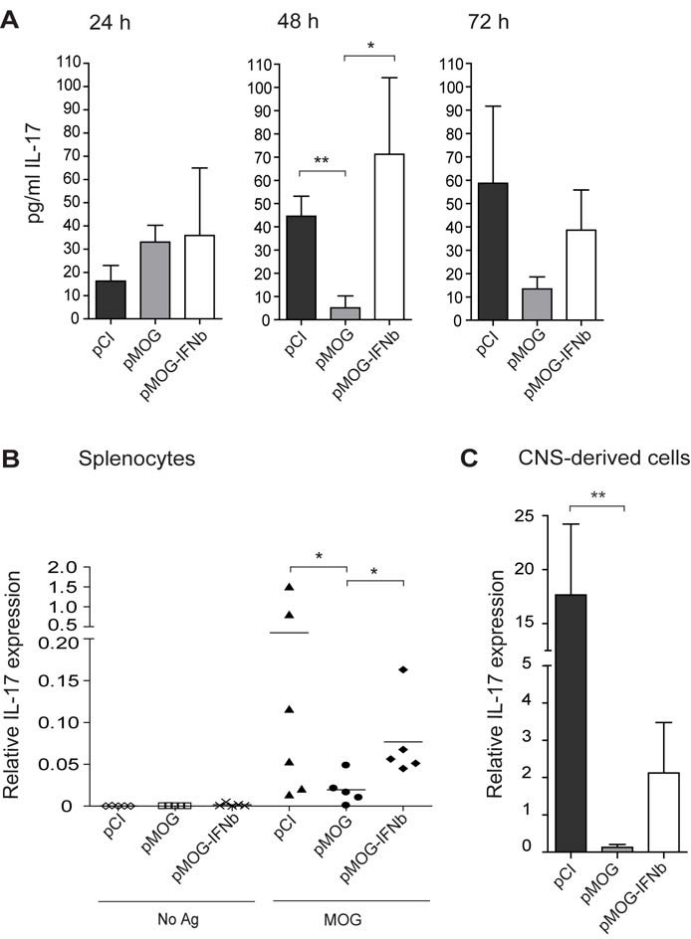
IL-17 responses are rescued by silencing of IFN-β during DNA vaccination. (A) Much lower IL-17 levels in supernatants from pMOG-vaccinated rats. Mean IL-17 protein levels in supernatants from splenocytes after 24 h, 48 h or 72 h culture with MOG_91–108_ (MOG) isolated from pMOG-, pMOG-IFNbeta- or pCI-treated rats, respectively, 11 d after MOG_91–108_ immunization (*n* = 7/group). Bars represent mean+/−SEM. (B) Mean IL-17 mRNA expression in splenocytes after 48 h culture with medium (No Ag) or MOG_91–108_ (MOG) isolated from pMOG-, pMOG-IFNbeta- or pCI-treated rats, respectively, 11 d after MOG_91–108_ immunization (*n* = 6/group). Data are representative of three separate experiments. All values are normalized to GAPDH mRNA. (C) Mean IL-17 mRNA expression in CNS-derived lymphocytes isolated from pMOG-, pMOG-IFNbeta- or pCI-treated rats (*n* = 7/group) at 11 d after MOG_91–108_ immunization. All values are normalized to GAPDH mRNA. Bars represent mean values. * *p*<0.05, ** *p*<0.01.

pMOG vaccination impaired Ag-specific IL-17 mRNA expression. To confirm a role for IFN-β during DNA vaccination on subsequent IL-17 responses we measured the levels of IL-17 protein in supernatants from splenocytes after 24 h, 48 h or 72 h culture with MOG_91–108_ isolated from pMOG-, pMOG-IFNbeta- or pCI-treated DA rats during peak of disease. The levels of IL-17 were strongly reduced in supernatants from pMOG-treated rats compared to pCI-treated controls after 48 h restimulation with MOG_91–108_ (*p*<0.01) (Fig. 7A). Importantly, the levels of IL-17 in supernatants from pMOG-IFNbeta-treated rats reached the same levels as in supernatants from pCI-treated controls after 48 h and 72 h restimulation, and were significantly higher than in supernatants from pMOG-treated rats after 48 h restimulation (*p*<0.05) (Fig. 7A).

IL-17 mRNA expression was higher (*p*<0.05) in splenocytes from pMOG-IFNbeta-treated than pMOG-treated rats (Fig. 7B). However, IL-17 expression in pMOG-IFNbeta-treated rats did not increase to the levels of pCI-treated rats although the difference between the groups was not significant (Fig. 7B). Ag-specific IL-21 expression did not differ between the groups (data not included). The experiment was repeated twice with the same results.

Finally we measured IL-17 mRNA expression in CNS-derived lymphocytes isolated during peak of disease from DNA vaccinated, pMOG-, pMOG-IFNbeta- or pCI-treated rats, respectively. Compared to pCI-treated controls, IL-17 mRNA expression was nearly absent in pMOG-treated rats (*p*<0.01) (Fig. 7C). The mRNA expression of IL-17 was twenty times higher in pMOG-IFNbeta-treated rats compared to pMOG-treated rats, although the levels did not reach the levels of pCI-treated rats (Fig. 7C). IL-21 mRNA expression was not induced in pMOG-IFNbeta-treated rats compared to pMOG-treated rats (data not included).

These data suggest that IFN-β mediates the DNA vaccine-conferred downregulation of IL-17 responses in the spleen, and is a likely explaination why pMOG-IFNbeta vaccination does not protect against EAE. The observed differences between protein and mRNA levels in splenocytes from pMOG-IFNbeta-treated rats may be caused by expansion of another cell type during restimulation in this group which would reduce the mRNA expression relative to housekeeping genes. This issue can be specifically addressed as soon as anti-rat IL-17 antibodies for intracellular staining becomes available.

Because IL-27 has been implicated in the mechanism of IFN-β- mediated suppression of autoimmunity and Th17 cell responses [28] we investigated the mRNA expression of IL-27p28 in MOG_91–108_-stimulated spenocytes from pMOG-, pMOG-IFNbeta- or pCI-treated rats. Unexpectedly, we observed reduced IL-27p28 expression in pMOG-treated mice compared to in pCI-treated mice (p<0.01) (data not included). This suggests that the suppressive effect of pMOG is not mediated by IL-27.

### Reduced numbers of CD4 Foxp3^+^ T cells following pMOG-IFNbeta vaccination

**Figure 8:**
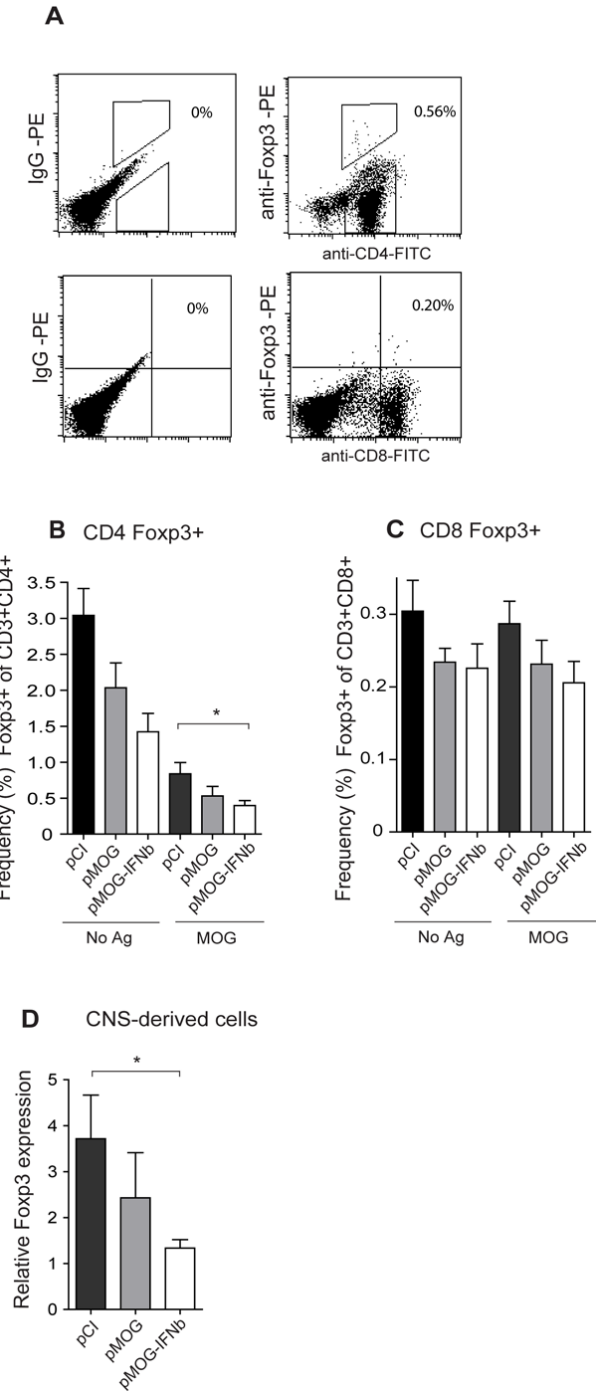
Reduced frequency of CD4 Foxp3^+^ T cells in spleen after pMOG-IFNbeta vaccination. (A) Percentage of CD4 Foxp3^+^ of total CD3^+^CD4^+^ T cells and CD8 Foxp3^+^ of total CD3^+^CD8^+^ T cells in spleen 11 d after MOG_91–108_ immunization. Mean frequency (%) of (B) Foxp3^+^CD4^+^CD3^+^ or (C) Foxp3^+^CD8^+^CD3^+^ T cells in spleen after 48 h culture with medium (No Ag) or MOG_91–108_ (MOG) isolated from pMOG-, pMOG-IFNbeta- or pCI-treated rats respectively (*n* = 7/group). (D) Mean Foxp3 mRNA expression in CNS-derived lymphocytes isolated from pMOG-, pMOG-IFNbeta- or pCI-treated rats (*n* = 7/group) at 11 d after MOG_91–108_ immunization. All values are normalized to GAPDH mRNA. Bars represent mean+/−SEM. * *p*<0.05.

We observed reduced Foxp3 mRNA expression following pMOG vaccination. To assess CD4 Foxp3^+^ and CD8 Foxp3^+^ T cell responses the frequency of Foxp3^+^ of total CD4^+^CD3^+^ or CD8^+^CD3^+^ T cells was measured by flow cytometry in splenocytes stimulated with or without MOG_91–108_ from pMOG-, pMOG-IFNbeta- or pCI-treated rats during peak of disease (Fig. 8A). The observed auto fluorescence was caused by the restimulation *ex vivo* and could not be further reduced by gating of the cells. The mean frequency of CD4 Foxp3^+^ T cells were reduced for all three groups after restimulation with MOG_91–108_. This is likely caused by clonal expansion of Ag-specific Th17 and Th1 cells *ex vivo*, which reduces the frequency of other Th cell types. It also suggests that induced, Ag-specific Treg are not induced following MOG_91–108_ immunization. The mean frequency of CD4 Foxp3^+^ T cells was not increased in pMOG-treated rats, but was reduced in pMOG-IFNbeta-treated rats compared to pCI-treated rats (p<0.05) (Fig. 8B).

The mean frequency of CD8 Foxp3^+^ T cells was not increased in either pMOG-treated or pMOG-IFNbeta-treated rats compared to in pCI-treated rats (Fig. 8C). In fact there was a tendency towards a decreased frequency of CD8 Foxp3^+^ T cells in both pMOG- and pMOG-IFNbeta treated rats (Fig. 8B and C).

We then measured Foxp3 mRNA expression in CNS-derived lymphocytes isolated during peak of disease from pMOG-, pMOG-IFNbeta- or pCI-treated rats, respectively. Foxp3 mRNA expression was lower in pMOG-IFNbeta-treated rats compared to in pCI-treated controls (*p*<0.05) (Fig. 8D).

Taken together these data suggest a pMOG-IFNbeta-conferred downregulation of CD4 Foxp3^+^ T cell responses in the spleen, and a tendency towards reduced CD4 Foxp3^+^ T cell responses in pMOG-treated rats.

## Discussion

We have previously studied Th responses subsequent to DNA vaccination but have been unable to link altered Th1 or Th2 responses to the capability of the DNA vaccine to protect from EAE development. Herein we report that Th17 cell responses during the peak of disease are dramatically impaired in DNA vaccinated rats compared to in controls. This may explain why DNA vaccination suppresses EAE, since Th17 cells mediate EAE [15], although Th1 cells also have a role in spinal cord parenchymal inflammation [17]. The suppressive effect of DNA vaccination was Th17 cell-specific, as only IL-17 and IL-21 responses were dampened in the CNS, and the expression of other relevant cytokines such as IFN-γ, IL-27, IL-6, IL-1β, TNF, IL-4 and IL-10 were not affected by DNA vaccination [3], [7], [11]. IL-17 and IL-21 are both expressed by Th17 cells but exert different functions, IL-17 being thought to be an effector molecule whereas IL-21 potentiates the Th17 cell response [29], [30].

Th17 and induced Foxp3^+^ Treg cells are activated through reciprocal mechanisms *in vitro* [16], [20] although further studies on the activation of these cells *in vivo* is warranted. The reduced Th17 cell responses reported herein could be caused by enhanced activation of induced Treg during DNA vaccination. We unexpectedly recorded a tendency towards reduced numbers of CD4 Foxp3^+^ and CD8 Foxp3^+^ T cells in the spleen following DNA vaccination compared to in controls during peak of EAE. Previous studies by us have demonstrated that the frequency of splenic CD4^+^CD25^+^ or CD4^+^CD25^hi^ Treg are likewise not affected by pMOG vaccination [7]. This is an important observation as it suggests that DNA vaccination dampens the Th17 cell response via mechanisms other than enhancing CD4 Foxp3^+^ or CD8 Foxp3^+^ T cell responses in our system. Probably the differentiation of Th17 cells is specifically impaired because Th1 and Th2 responses do not correlate with protection in our model [3], [7], [11]. In contrast, CD4^+^CD25^+^ Treg are implicated in the protective mechanism of DNA vaccination against experimental autoimmune uveitis [18] which suggests that the role of Treg during DNA vaccination differs between the different disease models and/or immunization regimens.

Guo, *et al* recently demonstrated that IFN-β-treated macrophages secrete IL-27, which in turn suppresses Th17 responses *in vitro* [28]. In contrast, we observed reduced IL-27 responses after DNA vaccination, which is in agreement with our previous findings that IL-10 is not enhanced by the pMOG vaccination [3], [7], [11]. Critical differences may be that we study suppression of Th17 responses *in vivo* whereas Guo *et al* study these responses *in vitro*; that IL-27 is expressed at an earlier timepoint in our system or that we use different species. Furthermore, pMOG-induced IFN-β may excert its function at a local site where few macrophages are present *in vivo*. Further analyses of T cell responses in DNA vaccinated rats are warranted and are ongoing in our laboratory.

The suppressive effect of pMOG is dependent on CpG DNA in the plasmid backbone and is associated with early induction of Interferon (IFN)-β [3], [7], [11]. However, the pathways that are activated by the DNA vaccine and which led to impaired Ag-specific Th17 immune responses have not been elucidated. Our microarray analyses demonstrated type I IFN-associated genes to be linked to protection following EAE induction. A specific, small set of IFN-inducible genes including IRF-7 and CCL19 were upregulated. This is in agreement with our previous studies in which IFN-β expression was enhanced following DNA vaccination [7] as well as a study in IFN-β-treated MS patients using microarrays that revealed upregulation of genes with IFN-responsive promoter elements but no alterations in Th1- or Th2-associated genes [31]. Similar to our microarray analyses, results from microarray analyses of peripheral blood from Systemic Lupus Erythematosus (SLE) patients suggest enhanced type I IFN production [32]. Moreover, plasma from SLE patients have elevated levels of circulating DNA enriched in hypomethylated CpG DNA [33]. Chromatin-IgG immune complexes (IC) isolated from these patients can induce pDC to produce high levels of IFN-α, and IC can also activate rheumatoid factor, B cells and dendritic cells [34]. In marked contrast, TLR9-deficient lupus-prone mice exhibit more severe lupus and activated pDC [35], [36], which concords with our previous findings that TLR9 and CpG DNA is linked to protection from autoimmunity [3], [11], [37].

Because we have previously observed enhanced IFN-β expression following DNA vaccination [3], [11] and type I IFN can inhibit differentiation of naïve CD4 T cells into Th17 cells *in vitro* [38], we studied the role of IFN-β during priming of Ag-specific T cells after DNA vaccination. The expression of IFN-β was specifically silenced both *in vitro* and *in vivo*, resulting in an abolished protective effect of the DNA vaccine construct. Our data demonstrates a requirement for early, local production of IFN-β during initiation of the suppressive immune response following DNA vaccination against EAE. We have thus unravelled a direct link between IFN-β exposure to T cells *in vivo* and subsequent suppression of EAE.

We investigated how IFN-β influences the Th17 and Foxp3^+^ T cell responses during DNA vaccination. Importantly, splenic IL-17 protein expression significantly developed in rats injected with the IFN-β-silencing DNA vaccine, pMOG-IFNbeta, compared to the suppressive DNA vaccine pMOG. However, IL-17 mRNA levels of expression did not reach the levels of the control rats. The reason for this could be: a) clonal expansion of another cell type in the pMOG-IFNbeta-treated group that skews the IL-17 mRNA expression relative to a housekeeping gene, or b) there are additional, unknown mechanisms involved. At present we lack tools to further dissect the effect of silencing of IFN-β on IL-17 expression. The numbers of CD4 Foxp3^+^ T cells were signifiantly reduced after silencing of IFN-β. We thus demonstrate a role for IFN-β in the suppression of Th17 responses during vaccination with pMOG.

Even though we have previously demonstrated a requirement for CpG DNA, TBK-1 phophorylates IRF-7 and IRF-3 and induces IFN-β via an unknown sensor of B form DNA such as plasmid DNA [12]. We speculate that TLR9 and TBK-1 act in synergy and are both required for the protective effect to occur following DNA vaccination. We propose a model for the protective mechanism of DNA vaccination against EAE that links immune reactivity towards plasmid DNA with suppression of MOG-specific Th17 cell responses and clinical signs of EAE. The expression of IFN-β is upregulated in response to DNA vaccine-derived B form DNA and/or CpG DNA. MOG_91–108_ expressed by the DNA vaccine is processed and presented on MHC II by dendritic cells to T cells. Enhanced IFN-β expression, in concert with unknown mechanisms, leads to failure of T cells to differentiate into pathogenic Th17 cells after subsequent MOG_91–108_-immunization, whereas the Th1 and Th2 responses remain intact [3].

In conclusion, we demonstrate that DNA vaccination downregulates Ag-specific Th17 cell responses, and that the suppressive capability of the DNA vaccine can be abrogated by silencing IFN-β.

## Materials and Methods

### Antigens

Peptide SDEGGYTCFFRDHSYQEE from rat sequence MOG_91–108_ was synthesized as previously described [3].

### Rats

All animal studies were reviewed and approved by the local ethical committee in Stockholm and Uppsala (Approval numbers C272/4 and C21/7, permission given to A. Lobell). Four-to-five wk old locally bred LEW.1AV1 (RT1^av1^) or DA (B&K, Sweden) female rats were used in the experiments.

### Plasmid construction

#### pMOG and pCI

Construction of pMOG (previously named pMOG_91–108_) and pCI were as previously described [3]. Briefly, seven tandem repeats of DNA coding for MOG_91–108_ were cloned into pCI (Promega, Madison, WI) to create pMOG. pCI, the plasmid backbone, is used as a control for pMOG.

#### pMOG-IFNbeta

Oligonucleotides coding for short hairpin RNA consisting of the sense strand of siRNA specific for IFN-β, a loop sequence, and the antisense stand of siRNA specific for IFN-β were hybridized and ligated into pSilencer 1.0 (Ambion, Austin, TX) directly downstream of a murine RNA polymerase U6 promoter, (sense 5′- GCACTAGCATTCGGACATGTTCAAGAGACATGTCCGAATGCTAGTGCTTTTTT -3′ and antisense 5′- AATTAAAAAAGCACTAGCATTCGGACATGTCTCTTGAACATGTCCGAATGCTAGTGCGGC C -3′). During RNA transcription from this sequence, one short hairpin RNA will form that is cleaved by Dicer intracellularly to generate an anti-IFN-β siRNA [39]. A BamHI-BamHI-fragment consisting of the U6 promoter/anti-IFN-β siRNA fragment was ligated into the plasmid backbone of a Bam HI-cleaved DNA vaccine, pMOG, encoding 7 repeats of the encephalitogenic MOG-peptide MOG_91–108_ to generate pMOG-IFNbeta.

#### pMOG-scr

As a negative control for the anti-IFN-β silencing by pMOG-IFNbeta, a siRNA-DNA vaccine was generated that consisted of a scrambled siRNA sequence without any specificity to any known RNA sequences in the rat: bp at three positions within the siRNA-coding portion of the siRNA were switched with the bp next to it, generating an siRNA that differed from the anti-IFN-β siRNA at six bp positions but had identical bp composition. Oligonucleotides coding for the sense strand of a scrambled siRNA sequence, a loop sequence, and the antisense strand of a scrambled siRNA sequence were hybridized and ligated into pSilencer 1.0 directly downstream of a murine RNA polymerase U6 promoter, (sense 5′- GACCTACGATTCGAGCATGTTCAAGAGACATGCTCGAATCGTAGGTCTTTTTT -3′ and antisense 5′-AATTAAAAAAGACCTACGATTCGAGCATGTCTCTTGAACATGCTCGAATCGTAGGTCGGCC -3′). A BamHI-BamHI-fragment consisting of the U6 promoter and a scrambled siRNA fragment was ligated into the plasmid backbone of a Bam HI-cleaved DNA vaccine, pMOG, to generate pMOG-scr.

### Plasmid preparation

Plasmid DNA was prepared using the Qiagen plasmid preparation protocol. Endotoxins were removed in an additional step (Endofree buffer set; Qiagen, Santa Clarita, CA).

### Transfection of cell lines

Rat marrow stromal cells were cultured to 50% confluence as previously described [27]. 10^5^ cells were transfected with 1 µg of pMOG-scr, pMOG-IFNbeta or PBS (Mock) in 7.5 µl of Super transfect reagent (Qiagen) according to the manufacturer's instructions and cultured for 48 h at 37 C.

### Western blotting

Cell lysates from transfected rat marrow stromal cells were subjected to SDS-PAGE (Novex pre-cast gels; Invitrogen life technologies, Carlsbad, CA) and transferred to nitrocellulose membranes (BioTrace®NT, PN 66485, Pall Life Sciences, Ann Arbor, MI). Membranes were incubated for 1 h in blocking buffer (TBS-0.2% Tween 20 with 5% w/v nonfat dry milk and 5% w/v BSA) then further incubated with a rat polyclonal anti-MOG_91–108_ antiserum diluted 1/100 in blocking buffer over night at 4°C. After incubation with a HorseRadish Peroxidase-conjugated goat-anti-rat Ab (Amersham Pharmacia Biotech, UK) for 1 h at R.T., the protein-Ab complexes were detected using ECL (Amersham Pharmacia Biotech, Uppsala, Sweden).

### Transfection of splenocytes

Rat splenocytes were isolated from five-to-six wk old female DA rats. 10^6^ cells were transfected with 0.5 µg of pMOG-scr, pMOG-IFNbeta or 1 ul H_2_O (Mock) and 0.5 µl PLUS reagent in 3 µl of Lipofectamine LTX (all from Invitrogen, Gaithersburg, MD) according to the manufacturer's instructions. Cells were cultured in DMEM supplemented with 10% heat inactivated fetal calf serum, 1% pencillin-streptomycin and 1% L-glutamine (all from Invitrogen) for 24 h at 37 C.

### Plasmid DNA injections and cardiotoxin pretreatment

Four-to-five wk old LEW.1AV1 or DA female rats were injected with 100 µl of 10 µM cardiotoxin (Latoxan, Rosans, France) in the gastrocnemii muscles. Seven d later rats were injected with 800 µg DNA at 2.0 mg/ml in PBS, divided into four 100 µl injections administered in the tibialii and gastrocnemii muscles, of either pCI, pMOG, pMOG-IFNbeta or pMOG-scr, respectively.

### EAE induction and clinical evaluation

Three wks after DNA vaccination rats were injected s.c. in the base of the tail with 200 µl inoculum containing 1∶1 100 µg MOG_91–108_ in PBS emulsified in CFA, consisting of IFA (Sigma, St Louis, MO) and 0.5 mg heat-inactivated *Myobacterium tuberculosis* (H37 RA strain, Difco Laboratories, Detroit, MI). The symptoms were scored as follows: grade 1; tail weakness or tail paralysis, grade 2; hind leg paraparesis, grade 3; hind leg paralysis, grade 4; complete paralysis, moribund state or death.

### Splenocyte preparation and culture

Spleens from DNA vaccinated and MOG_91–108_-immunized rats were disrupted and cells were suspended in DMEM (Invitrogen). Mononuclear cells were resuspended in DMEM supplemented with 10% heat inactivated fetal calf serum, 1% pencillin/streptomycin and 1% L-glutamine (all from Invitrogen), and flushed through a 70 µm plastic strainer (Becton Dickinson, Mountain View, CA), adjusted to 2×10^6^ cells/ml, and cultured with or without 10 µg/ml MOG_91–108_ for 24, 48 or 72 h at 37 C in a humidified atmosphere containing 5% CO_2_.

### CNS-derived lymphocyte isolation

Lymphocytes were isolated from brain and spinal cord from pMOG, pMOG-IFNbeta or pCI vaccinated and MOG_91–108_ immunized rats 11 days after immunization as previously described [3].

### Isolation of T cells

CD3^+^ T cells were purified from MOG_91–108_-stimulated splenocyte cultures using CD3-MACS magnetic beads according to the manufacturer's instructions (Miltenyi Biotec GmbH, Bergisch Gladbach, Germany) and CD3^+^CD4^+^ Th cells were sorted by flow cytometry to 90% purity. The following antibodies were used for staining: anti-CD3-APC and anti-CD4-FITC (all from BD Biosciences). Sorted cells were frozen in −70 C for subsequent RNA isolation, cDNA synthesis and Q-PCR.

### Intracellular staining of Foxp3 in T cells

Splenocytes from DNA vaccinated and MOG_91–108_-immunized rats were cultured with or without MOG_91–108_ for 48 h. Cells were fixed, permeabilized and incubated with 1% normal rat serum to prevent non-specific binding of antibodies. The following antibodies were used for staining: anti-CD3-APC (BD Biosciences), anti-Foxp3-PE (Biolegend, San Diego, CA) and anti-CD4-FITC (Biolegend) or anti-CD8b-FITC (Biolegend). Mouse IgG1-PE was used as isotype control (Biolegend). Cells were analyzed on a FACSCalibur™ flow cytometer (BD Biosciences) using Cellquest software (BD Biosciences).

### Supernatant IL-17 ELISA

Rat IL-17 was measured in supernatants from MOG_91–108_-stimulated splenocyte cultures by ELISA according to manufacturer's instructions (USCN Life Science and Technology Company, China). The lower limit of detection is 3.9 pg/ml for IL-17.

### Quantification of mRNA expression

Q-PCR to quantify levels of cytokines has been previously described [40], [41]. RNA isolation and subsequent cDNA preparation were performed as previously described [40]. Quantitative analyses of mRNA expression were performed using QuantiTect™ SYBR® green according to the manufacturer's instructions (Qiagen) and amplification was performed using an ABI prism 7700 Sequence Detection System (ABI, Norwalk, CT) or MyiQ Cycler (Bio-Rad laboratories). Samples were analyzed as previously described [7]. PCR products were visualized by electrophoresis in 4% Et-Br containing agarose gels.

### cDNA microarray analysis

The generation, use and analysis of microarrays representing 6240 cDNAs has been described previously [42]–[44]. The guidelines and checklist of MIAME (www.mged.org/workgroups/MIAME) were followed. cDNA clones were selected from the TIGR rat gene index (www.tigr.org) or purchased from Research Genetics (Invitrogen Carlsbad, CA). The platform is available online and is designated GLP541 at Gene Expression Omnibus (GEO) at www.ncbi.nlm.nih.gov/geo. Total RNA from pMOG91–108- vaccinated rats or pCI-treated control rats were isolated from flash-frozen spleens 11 d after EAE induction using RNeasy maxi RNA isolation kit (Qiagen). cDNA from three individual DNA vaccinated rats was labelled with Cy5, and cDNA from three individual control rats was labelled with Cy3. First, each Cy5-labelled DNA vaccine cDNA was compared with one of the Cy3-labelled control cDNA in a hybridisation. The three DNA vaccine cDNAs were then labelled with Cy3 and the three control cDNAs were labelled with Cy5 and hybridised to the microarray. Labelling and hybridization protocols have been previously described [43]. The data series is available online at GEO at www.ncbi.nlm.nih.gov/geo, is designated GSE1538 and consists of the six samples GSM26467-GSM26472.

### Statistical analysis of microarray data

Data was normalized by Lowess normalization using the statistical software R. The significance of the expression ratios was then estimated using the Significance Analysis of Microarray (SAM) statistical technique [45]. A *q* value was assigned for all detectable genes. The *q* value represents the lowest false discovery rate at which the differential expression of the gene is considered significant. Only genes with a *q* value of less than 5% were considered significantly differentially expressed. In an additional step, only genes with a ratio of 1.4 or higher were considered. Then the genes were categorized using Gene Ontology annotations program (http://apps1.niaid.nih.gov/David/gochart). Because rat genes are not as well annotated as human genes, the rat gene identities were mapped to human locuslink numbers of orthologues genes. These were then used to search in the GOchart program.

### Isolation and culture of monocytes

Monocytes were obtained from heparinized blood of 4–5 rats using standard Lymphoprep density gradient centrifugation (Nycomed, NY). Monocytes were further enriched by magnetic beads conjugated to RT1 Ab (Miltenyi Biotec, Germany) following the instructions provided by the manufacturer. Subsequent to this procedure approximately 90% of the cells were MHC class II^+^ as assessed by flow cytometry. Cells were plated into 6 well plates (Nunc, Roskilde, Denmark) at a concentration of 1–2×10^6^ cells/well in 1 ml DMEM supplemented with 10% heat inactivated fetal calf serum, 1% pencillin-streptomycin, 1% L-glutamine (all from Invitrogen) with GM-CSF (5 ng/ml, R&D Systems, Oxon, UK) and IL-4 (25 ng/ml, R&D systems) or GM-CSF and IFN-β (1000 U/ml a kind gift from Peter van Der Meide). After three d cells were harvested and used for further analysis.

### Statistical analysis

Differences between mean daily and accumulated EAE scores were analyzed with Mann Whitney's U test. *p* values lower than 5% were considered significant. To measure differences between gene expression and cytokine levels, we first tested if the groups were normally distributed. If they were, we analyzed differences using unpaired t-test. If the groups were not normally distributed, we analyzed the differences with Mann-Whitney U test or Kruskal-Wallis test to compare three or more groups using Graphpad Prism 4.0 software. *p* values lower than 5% were considered significant.
